# Association of increased basic salivary proline‐rich protein 1 levels in induced sputum with type 2‐high asthma

**DOI:** 10.1002/iid3.602

**Published:** 2022-03-10

**Authors:** Fengjia Chen, Yuxia Liang, Zhimin Zeng, Lijuan Du, Changyi Xu, Yubiao Guo, Canmao Xie

**Affiliations:** ^1^ Division of Pulmonary and Critical Care Medicine The First Affiliated Hospital of Sun Yat‐sen University Guangzhou China

**Keywords:** asthma, induced sputum, phenotype, PRB1

## Abstract

**Background:**

The aim of this study is to reveal whether basic salivary proline‐rich protein BstNI subfamily 1 (PRB1) may be used as a diagnostic biomarker for type 2‐high asthma.

**Methods:**

PRB1 protein levels in the induced sputum of 67 subjects with asthma and 27 controls were determined by an enzyme‐linked immunosorbent assay. Correlation analyses between PRB1 in the induced sputum and airway inflammatory indicators were also performed.

**Results:**

PRB1 protein levels were significantly upregulated in the induced sputum of asthmatic patients (*p* =0.0098) and correlated with clinical eosinophil‐related indicators and type 2 airway inflammation. These results indicate that PRB1 is a promising biomarker for type 2‐high asthma.

**Conclusions:**

The expression of PRB1 in induced sputum is a potential biomarker for type 2‐high asthma. The results of this study present new insights into the diagnosis and treatment of asthma.

## Graphical abstract

Our study suggests that proline‐rich protein BstNI subfamily 1 (PRB1) expression in induced sputum is increased in asthmatic subjects, which demonstrates its association with type 2‐high asthma. Thus, PRB1 may be a biological marker for type 2‐high asthma. The findings of this study could provide new insights into the diagnosis and treatment of asthma.

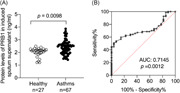

## 1 INTRODUCTION

Asthma, which is characterized by airway hyperresponsiveness, airway eosinophilic inflammation, mucus overproduction, and airway remodeling, is gradually becoming an increasingly common disease. Airway eosinophilic inflammation is a central feature of asthma.^1^, ^2^, ^3^ Asthma is a heterogenous disease with different phenotypes, including type 2‐low and type 2‐high asthma. T‐helper type 2‐driven inflammation defines the major subphenotypes of asthma featuring airway eosinophilic inflammation.^4^, ^5^ Sputum induction, a repeatable, noninvasive method to detect direct responses to airway inflammation, plays a critical role in determining the inflammatory phenotype of asthma.^6^, ^7^ The discovery of biomarkers in induced sputum may help identify asthma phenotypes and therapeutic targets toward a better understanding of asthmatic airway inflammation.^8^

In our preliminary study, we performed transcriptome microarray analysis on four pairs of bronchial brush specimens from asthmatics and healthy controls, and found that basic salivary proline‐rich protein (PRP) BstNI subfamily 1 (PRB1) is a significantly upregulated molecule. We thus speculate that PRB1 may play a role in asthma. To confirm our hypothesis, we determined the protein levels of PRB1 in the induced sputum of symptomatic, recently diagnosed asthmatic and healthy subjects by enzyme‐linked immunosorbent assay (ELISA). We then performed correlation analyses between PRB1 protein levels and various airway inflammation indicators, to determine the function of PRB1 in asthma further.

## 2 METHODS

### 2.1 Subjects

We recruited 27 healthy control subjects and 67 symptomatic, recently diagnosed subjects with asthma from September 2020 to March 2021. All of the subjects were Chinese and recruited from the First Affiliated Hospital of Sun Yat‐Sen University, an academic teaching hospital. Subjects with asthma were diagnosed by a physician according to the definition of the Global Initiative for Asthma Criteria (GINA).^9^ The diagnostic criteria for asthma are as follows: the patient has symptoms of episodic cough, wheezing, and/or dyspnea, as well as airway hyperresponsiveness or airway reversibility. In our study, airway hyperresponsiveness was defined as inhaled histamine concentration ≤8 mg/ml and a 20% reduction in forced expiratory volume in the first second (FEV_1_) from baseline. The positive response of bronchial dilation test(BDT) was defined as an improvement of ≥12% in FEV_1_ and an increase in ≥200 ml following 400 μg inhalations of a salbutamol‐metered dose inhaler with a spacer.

Healthy control subjects had no respiratory symptoms or a history of other respiratory or immune system‐related diseases. None of the subjects had ever smoked or received inhaled or oral corticosteroids or leukotriene antagonists. The study was approved by the Institutional Research Ethics Committee of the First Affiliated Hospital of Sun Yat‐Sen University and signed informed consent was obtained from all subjects before their participation in this study.

### 2.2 Baseline evaluation

Demographic information and induced sputum samples were collected from each subject. Pulmonary function test(PFT), bronchial provocation test(BPT), or BDT and fraction of exhaled nitric oxide (FeNO) were measured upon study entry.

### 2.3 Measurements of FeNO, PFT, BPT, and BDT

#### 2.3.1 FeNO measurement

We used a NIOX MINO analytical instrument for FeNO detection (Aerocrine AB). The procedure was based on the recommendations of the American Thoracic Society/European Respiratory Society. The participants were instructed to inhale air without NO to total lung capacity and immediately exhale fully into the device at a constant expiratory flow rate of 50 ml/s for 10 s.^10^

#### 2.3.2 PFT, BPT, and BDT tests

We used a body box (Medi‐soft MODEL 5500) to perform the PFT, BPT, and BDT tests. PFT was assessed for each enrolled subject. Subjects with FEV_1_ (% predicted) of >70% were subjected to the BHR test to determine the presence of bronchial hyperresponsiveness and subjects with FEV1 (% predicted) of <70% were subjected to the BPT to determine airway reversibility. These tests followed the recommendations of the Chinese National Guidelines of Pulmonary Function Test.^11^ The PFT was repeated at least three times for each subject and the best value was selected from the acceptable results.

### 2.4 Collection of induced sputum

Subjects were asked to inhale atomized 4.5% sodium chloride solution to induce coughing and their sputum was collected. The sputum samples were processed within 2 h of collection. A sputum cell pellet was formed, weighed, and added with a volume of 0.1% DL‐Dithiothreitol amounting to four times its weight for dissolution. The solution was then filtered by cell sieving. After centrifugation, the sputum supernatant was separated for ELISA detection.

### 2.5 ELISA

Protein levels of PRB1 in the induced sputum supernatant were measured using a commercial ELISA according to the manufacturer's protocol (Shanghai Yubo Industrial, Human Basic Salivary Proline‐Rich Protein ELISA Kit, Catalog number YB‐PRB1‐Hu 96 Tests). Protein levels of interleukin (IL)‐2, IL‐4, IL‐5, IL‐6, IL‐10, IL‐13, IL‐17, interferon (IFN‐γ), Periostin (POSTN), mucin 5AC (MUC5AC), and serpin family B member 2 (SERPINB2) in the induced sputum supernatant were also measured using commercially available ELISA kits (MEIMIAN) according to the manufacturer's instructions.

### 2.6 Statistical analysis

Prism version 8 was used to analyze the data (GraphPad Software). We calculated means ± SDs and used the unpaired *t* test to compare groups. Spearman's rank‐order correlation was used to analyze correlations. The area under the curve (AUC) of the receiver operating characteristic (ROC) curve was used to evaluate the diagnostic value of PRB1 for differentiating asthma from healthy subjects. A *p* < .05 was considered statistically significant.

## 3 RESULTS

### 3.1 Subject characteristics

A total of 67 newly diagnosed asthma patients who met the inclusion criteria and 27 healthy controls were recruited to this study. The average age of the asthmatic patients was 43.09 ± 15.64 years and their level of FeNO was 57.45 ± 47.06 parts per billion. Compared with the healthy controls, asthmatic subjects had lower PFT parameters and higher levels of FeNO, blood eosinophils (EOS), and serum IgE. The characteristics of the subjects are detailed in Table 1.

**Tabel 1:** Clinical characteristics of subjects

Parameter	Asthma (*N* = 67)	Control (*N* = 27)	*p*
Gender (M/F)	39/28	10/17	0.0722
Mean age, years	43.09 ± 15.64	31.81 ± 13.49	0.0015
Height (cm)	166.5 ± 8.706	163.8 ± 7.689	0.1575
Weight (kg)	64.59 ± 11.15	56.54 ± 7.886	0.0008
BMI (kg/m^2^)	23.22 ± 3.083	21.82 ± 3.050	0.0453
FVC (% predicted)	92.48 ± 17.87	102.4 ± 10.94	0.0074
FEV_1_ (% predicted)	79.76 ± 25.60	102.8 ± 8.318	<0.0001
FEV_1_/FVC (%)	70.75 ± 14.74	86.59 ± 6.621	<0.0001
PEF (% predicted)	71.76 ± 25.18	79.54 ± 11.55	0.1213
MEF (% predicted)	60.79 ± 32.62	100.6 ± 18.33	<0.0001
MEF25 (% predicted)	55.07 ± 36.21	107.5 ± 29.80	<0.0001
MEF50 (% predicted)	59.14 ± 33.52	100.5 ± 23.19	<0.0001
MEF75 (% predicted)	63.22 ± 31.01	83.79 ± 13.49	0.0011
FeNO (ppb)	57.45 ± 47.06	13.87 ± 5.743	<0.0001
Total serum IgE (IU/ml)	249.9 ± 311.4	100.2 ± 106.1	0.0500
Blood eosinophil counts 10^9^/L	0.3239 ± 0.2933	0.1052 ± 0.045	0.0011
Blood eosinophil percentage (%)	4.676 ± 4.331	1.762 ± 0.8823	0.0031

*Note*: Measured pulmonary function values are presented as a predictive percentage. Data are shown as mean ± SD unless indicated otherwise.

Abbreviations: BMI, body mass index; F, female; FeNO, fractional exhaled nitric oxide; FEV1, forced expiratory volume in 1 s; FVC, forced vital capacity; M, male; MEF, maximal midexpiratory flow; MEF25, forced expiratory flow after 25% of the FVC; MEF50, forced expiratory flow after 50% of the FVC; MEF75, forced expiratory flow after 75% of the FVC; PEF, peak expiratory flow; ppb, parts per billion.

### 3.2 PRB1 levels in the induced sputum supernatant

**Figure 1:**
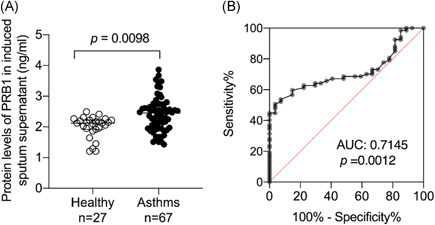
Proline‐rich protein 1 BstNI subfamily 1 (PRB1) levels in the induced sputum supernatant. (A) The protein levels of PRB1 in induced sputum supernatant were determined by enzyme‐linked immunosorbent assay (ELISA; 27 healthy controls vs. 67 asthmatic patients). (B) The receiver operating characteristic (ROC) curve of PRB1 in induced sputum supernatant

We conducted ELISA to analyze the protein levels of PRB1 in the induced sputum supernatant to detect the expression of PRB1 in asthma and found that PRB1 is increased in the induced sputum of the asthmatic group (*p* = 0.0098; Figure 1A). An ROC curve was constructed to discriminate asthma from healthy subjects. PRB1 levels in induced sputum supernatant are helpful in the diagnosis of asthma and the AUC was 0.7145 (*p* = 0.0012; Figure 1B).

### 3.3 Association of PRB1 protein levels in the induced sputum supernatant with clinical EOS‐related indicators

**Figure 2:**
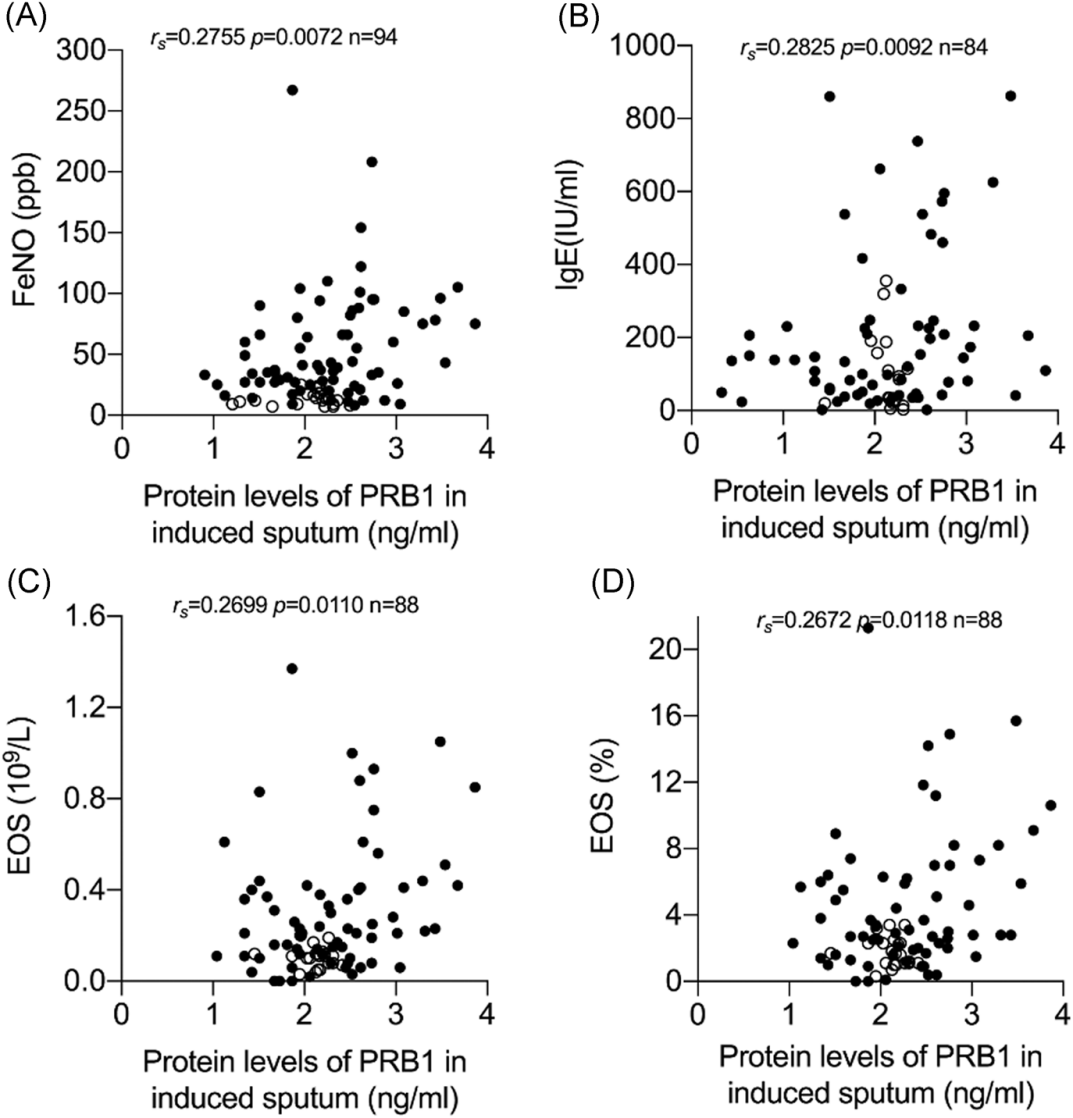
Association of proline‐rich protein 1 BstNI subfamily 1 (PRB1) protein levels in the induced sputum supernatant with clinical eosinophil‐related indicators. Spearman's rank‐order correlation assay between PRB1 protein levels in induced sputum supernatant and (A) FeNO, (B) total serum IgE, (C) blood eosinophil counts 10^9^/L, and (D) blood eosinophil percentage (%). Asthma patients were presented as dots and healthy controls as circles

To investigate the role of PRB1 in induced sputum supernatant and eosinophilic inflammation, we explored the correlation of PRB1 in the induced sputum supernatant with clinical EOS‐related indicators, such as FeNO, total serum IgE, blood EOS counts, and percentage of EOS in peripheral blood (EOS%). Results showed that protein levels of PRB1 in the induced sputum supernatant are positively correlated with FENO (*r*_s_ = 0.2755, *p* = 0.0074, *n* = 94), total serum IgE (*r*_s_ = 0.2825, *p* = 0.0092, *n* = 84), EOS (*r*_s_ = 0.2699, *p* = 0.0110, *n* = 88), and EOS% (*r*_s_ = 02672, *p* = 0.0118, *n* = 88; Figure 2A–D). These data suggest that PRB1 may be related to eosinophilic inflammation in asthma.

### 3.4 Correlation of PRB1 protein levels with cytokines related to type 2‐high asthma in induced sputum supernatant

**Figure 3:**
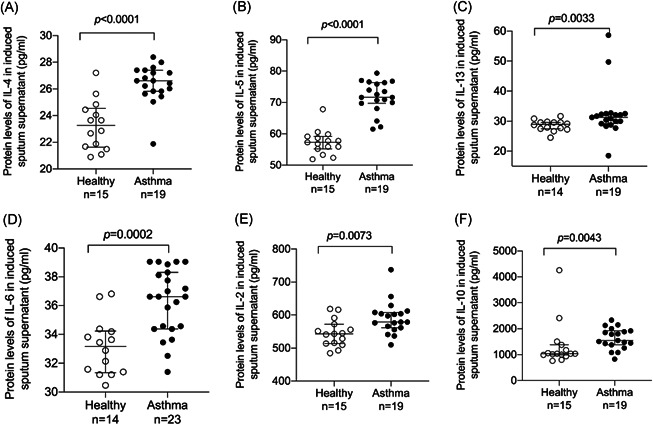
The levels of inflammatory cytokines were increased in induced sputum of the asthmatic group compared with that of the control group. The levels of inflammatory cytokines (A) interleukin (IL)‐4, (B) IL‐5, (C) IL‐13, (D) IL‐6, (E) IL‐2, and (F) IL‐10 in induced sputum were determined by enzyme‐linked immunosorbent assay (ELISA; healthy controls vs. asthmatic patients)

**Figure 4:**
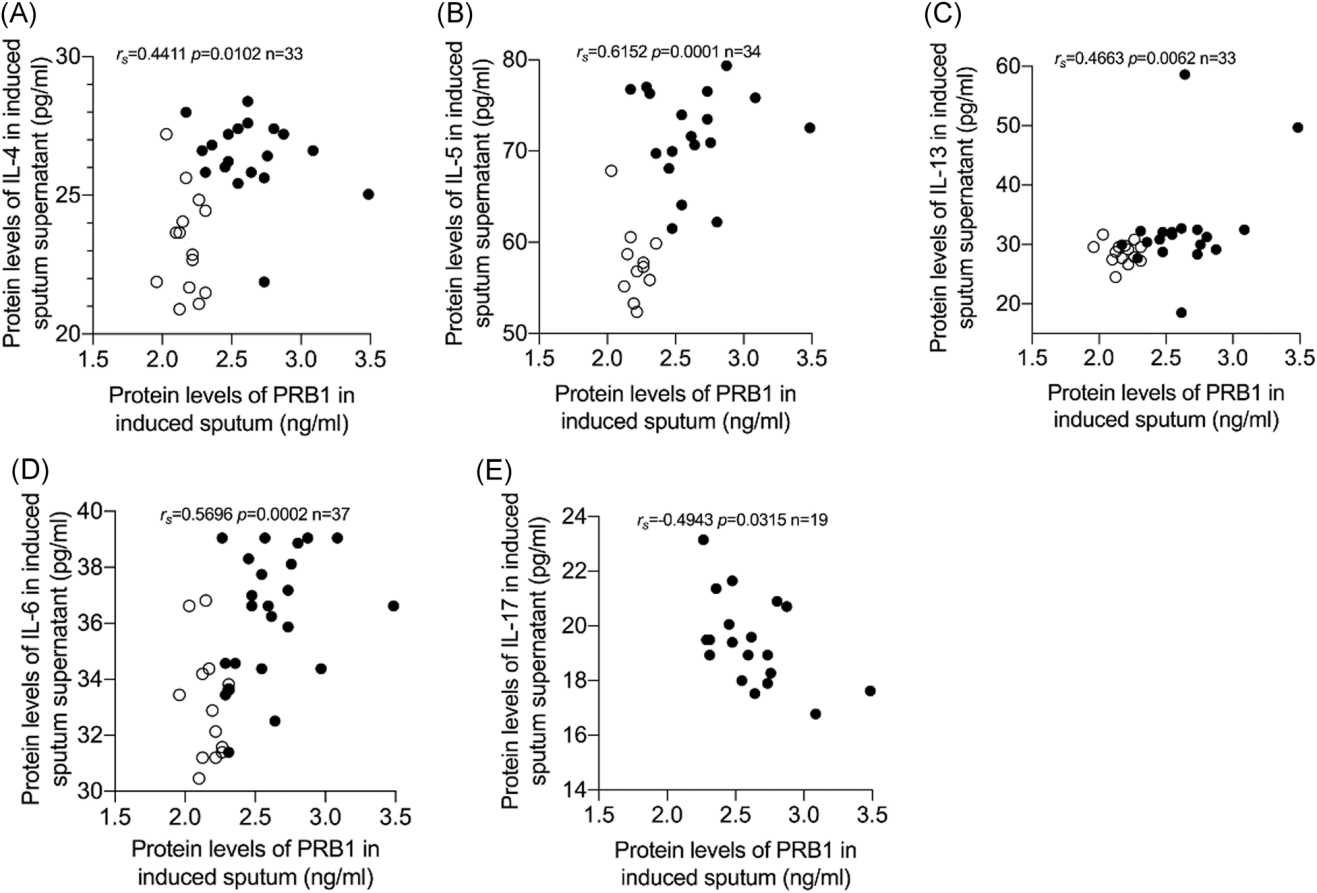
Correlation of proline‐rich protein 1 BstNI subfamily 1 (PRB1) protein levels with cytokines related to type 2‐high asthma in induced sputum supernatant. Spearman's rank‐order correlation assay between PRB1 protein levels and levels of (A) interleukin (IL)‐4, (B) IL‐5, (C) IL‐13, (D) IL‐6, and (E) IL‐17 in induced sputum supernatant. Asthma patients were presented as dots and healthy controls as circles

The levels of cytokines (IL‐2, IL‐4, IL‐5, IL‐6, IL‐10, IL‐13, IL‐17, and INF‐γ) in the induced sputum supernatant were determined by ELISA. IL‐4, IL‐5, IL‐13, IL‐6, IL‐2, and IL‐10 levels in the induced sputum of the asthmatic group were higher compared with those of the control group (Figure 3A–F). The levels of cytokines INF‐γ and IL‐17 showed no statistical significance between the two groups (Figure S1A,B). Protein levels of PRB1 in the induced sputum supernatant were significantly positively correlated with IL‐4, IL‐5, IL‐13, and IL‐6 (*r*_s_ = 0.4411, *p* = 0.0102; *r*_s_ = 0.6152, *p* = 0.0001; *r*_s_ = 0 .4663, *p* = 0.0062; and *r*_s_ = 0.5696, *p* = 0.0002, respectively; Figure 4A–D) and significantly negatively correlated with IL‐17 (*r*_s_ = −0.4943, *p* = 0.0315; Figure 4E). No significant correlation between protein levels of PRB1 and levels of cytokines IL‐2, IL‐10, and INF‐γ was noted (Figure S1C–E).

### 3.5 Correlation of PRB1 protein levels with proteins related to type 2‐high asthma in induced sputum supernatant

**Figure 5:**
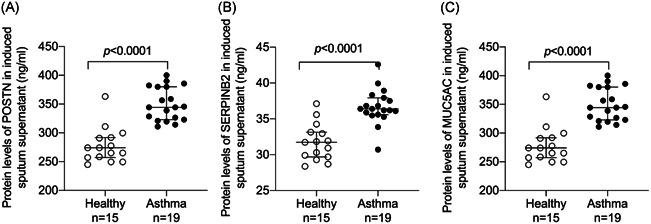
The levels of proteins related to type 2‐high asthma were increased in induced sputum of the asthmatic group compared with that of the control group. The levels of (A) POSTN, (B) SERPINB2, and (C) MUC5AC were increased in induced sputum of the asthmatic group compared with that of the control group

**Figure 6:**
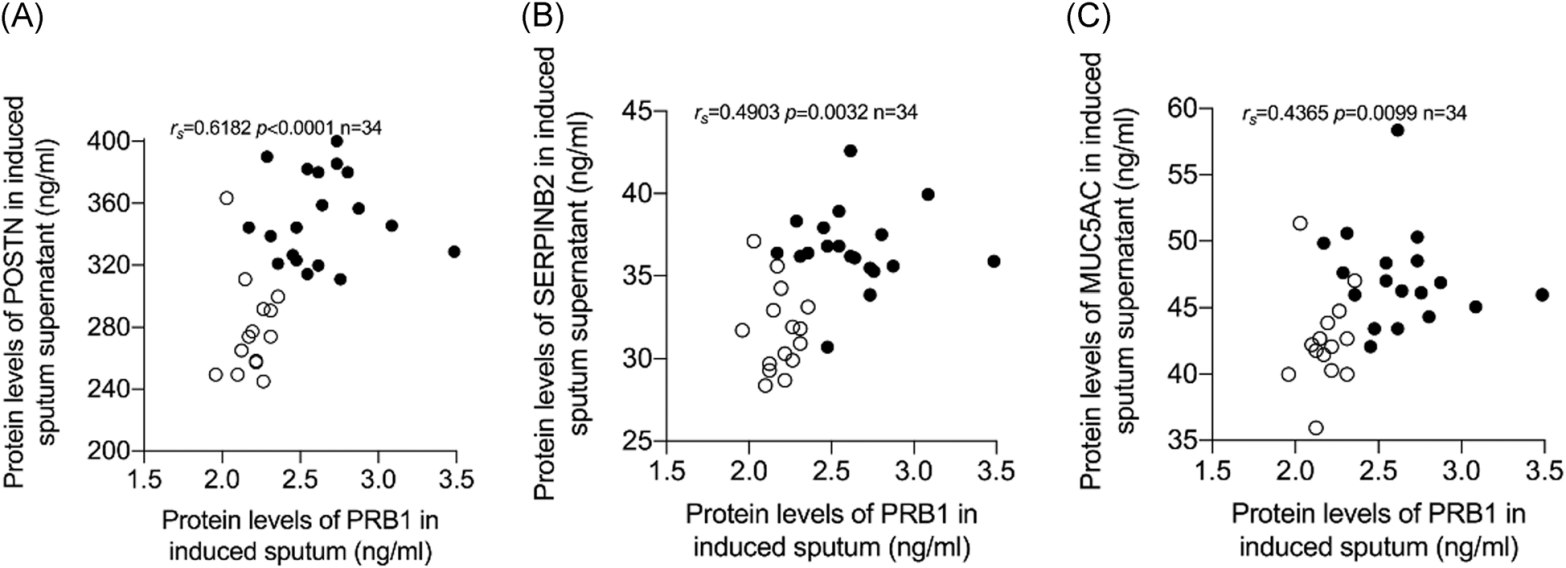
Correlation of proline‐rich protein 1 BstNI subfamily 1 (PRB1) protein levels with proteins related to type 2‐high asthma in induced sputum supernatant. Spearman's rank‐order correlation assay between PRB1 protein levels in induced sputum supernatant and levels of (A) POSTN, (B) SERPINB2, and (C) MUC5AC in induced sputum supernatant. Asthma patients were presented as dots and healthy controls as circles

The protein levels of POSTN, SERPINB2, and MUC5AC in the induced sputum supernatant were determined by ELISA. The levels of POSTN, SERPINB2, and MUC5AC in the induced sputum of the asthmatic group were higher compared with those in the control group (Figure 5A–C). Protein levels of PRB1 in the induced sputum supernatant were significantly positively correlated with the protein levels of POSTN, SERPINB2, and MUC5AC (*r*_s_ = 0.6182, *p* < .0001; *r*_s_ = 0.4903, *p* = 0.0032; and *r*_s_ = 0 .4365, *p* = 0.0099, respectively; Figure 6A–C).

## 4 DISCUSSION

To the best of our knowledge, this study is the first to demonstrate that protein levels of PRB1 are significantly upregulated in the induced sputum of asthmatic patients (*p* = 0.0098) and positively correlated with type 2 airway inflammation. Such findings suggest that PRB1 in induced sputum has potential diagnostic value for asthma and may be a promising biomarker for type 2‐high asthma.

Asthma is a heterogeneous immunopathological disease, with features of chronic inflammation, airway hyperresponsiveness, mucus over secretion, and airway remodeling.^1^, ^2^ Estimates of the global prevalence of asthma in different populations range from 1% to 18% and as many as 300 million people have asthma globally with increasing annual cost to the healthcare system.^12^ According to GINA, the diagnosis of asthma should be based on the patient's clinical symptoms combined with laboratory evidence of BPT or BDT. However, BPT is time‐consuming and has a high risk of inducing severe bronchospasm. At the same time, patients with acute exacerbation of asthma can not carry out lung function testing.^13^ Easy assessible and noninvasive collection of induced sputum made it reproducible and sensitive to be used for the assessment of asthma. Previous studies on PRB1 have focused on saliva, which may contribute to oral lubrication, maintenance of pH balance, and protection against microbes.^14^, ^15^, ^16^ As our results show, PRB1 protein significantly upregulated in the induced sputum of asthmatic patients, which could be used as a diagnostic tool for distinguishing asthmatic patients from healthy controls. To establish a predictive model to differentiate asthmatic patients from healthy controls, ROC curves were conducted to evaluate the importance of PRB1. We found that the area under ROC curve was equal to 0.7145, indicating PRB1 protein in induced sputum has diagnostic significance in asthma.

Asthma represents a complex, heterogeneous set of diseases with different phenotypes. Identifying the phenotype of asthma patients is important for individual therapy, especially when choosing targeted biological or immunomodulatory therapies; indeed, biomarkers are often needed to help distinguish between different phenotypes.^17^, ^18^ The pathobiology of asthma can be broadly classified by endotypes of type 2‐high and type 2‐low disease. Type 2‐high inflammation defines the major subphenotypes of asthma and airway eosinophilic inflammation is a key feature of type 2‐high asthma.^4^ Thus far, the gold standard for airway inflammation is bronchial mucosal biopsy. However, as it is an invasive procedure, bronchial mucosal biopsy is unlikely to be performed clinically. As airway epithelial cells play a key role in airway inflammation, we often use the induced sputum cell technique as a noninvasive method to assess airway inflammation.^19^

Human PRPs are determined by six closely linked genes on chromosome 12p13.2 and can be divided into two main families, namely type HaeIII and type BstNI.^20^ PRB1 belongs to the BstNI family. PRB1 is a protein‐coding gene that encodes a member of the heterogeneous family of basic, proline‐rich, human salivary glycoproteins.^15^ Alternative splicing results in multiple transcription variants encoding different isoforms with similar proteolytic processes.^21^ Chang et al.^22^ conducted a gene chip‐based bioinformatics analysis of allergic rhinitis (AR) using the Gene Expression Database to obtain the biomarkers of AR. The authors then collected the inferior turbinate mucosa tissues of 15 AR patients and 15 healthy controls for further verification and found that *PRB1* is one of the genes with the most obvious expression difference. However, this gene has yet to be studied extensively. Moreover, PRB1 has not been reported in asthma or other airway inflammatory diseases.

In the present study, PRB1 was significantly upregulated in the induced sputum of asthmatic patients, with an AUC of 0.7415. This finding suggests that PRB1 protein levels in induced sputum have good diagnostic value in distinguishing asthma from healthy subjects. Moreover, PRB1 expression was significantly positively correlated with EOS‐related indicators, such as FeNO, blood EOS, and serum IgE. FeNO detection is clinically used as a marker of airway inflammation associated with pathways mediated by the type 2 inflammatory cytokines IL‐4 and IL‐13. Elevated FeNO levels are associated with allergies and eosinophilic asthma, and thus could help identify patients with these phenotypes. Patients with AR can also show increased FENO.^23^ IgE, an important marker of airway inflammation, plays a key role in allergic asthma. The production of IgE requires a class switching within activated B cells that is induced by IL‐4 and IL‐13. Elevated IgE supports the clinical and inflammatory characteristics of type 2 inflammatory pathway activity.^4^, ^24^ Type 2‐high asthma also results in elevated peripheral blood or airway EOS. The number of EOS in peripheral blood is another biomarker for type 2‐high asthma.^24^, ^25^ Protein levels of PRB1 in induced sputum positively correlated with FENO, total serum IgE, EOS, and EOS% in peripheral blood, and these clinical indicators are biomarkers of type 2‐high asthma. This finding suggests that PRB1 may be a signature gene for type 2‐high asthma, in particular playing a key role in eosinophilic inflammation asthma.

To determine the relationship between PRB1 and type 2‐high asthma further, we detected the levels of IL‐2, IL‐4, IL‐5, IL‐6, IL‐10, IL‐13, IL‐17, and INF‐γ in the induced sputum supernatant. Compared with the control group, asthmatic patients showed increased levels of IL‐2, IL‐4, IL‐5, IL‐6, IL‐10, and IL‐13 in their induced sputum supernatant, and PRB1 protein levels in the sputum supernatant were positively correlated with IL‐4, IL‐5, and IL‐13, which are associated with type 2‐high asthma. No significant correlation between protein levels of PRB1 with levels of IL‐2, IL‐10, and INF‐γ, which are associated with type 2‐low asthma, were detected. Th2 cytokines, including IL‐4, IL‐5, and IL‐13, are important factors in the pathophysiological characteristics of allergic asthma.^26^ Non‐T2 cytokines, such as IL‐2, IL‐10, and IFN‐γ, have been implicated in the pathogenesis of asthma, particularly in patients with severe disease.^27^ IL‐17 promotes neutrophilic inflammation and is secreted by Th17 cells.^28^ Many studies have confirmed that IL‐17A is involved in the development of severe asthma, which is characterized by neutrophil infiltration in the airway. In the present study, no difference in IL‐17 level in the sputum supernatant between asthmatic patients and healthy controls was noted; this finding may be attributed to the fact that all asthmatic patients in our study were newly diagnosed and, therefore, not severe cases. IL‐17 can inhibit Th2 cytokine production and EOS recruitment.^29^ Although we found no statistically significant difference in IL‐17 level between asthma patients and the control group, protein levels of PRB1 were negatively correlated with IL‐17A in the induced sputum supernatant.

**SERPINB2 and POSTN are an epithelial gene signature for type 2‐high asthma.^18^, ^30^ During asthma progression, MUC5AC is specifically expressed in goblet cells. Mucus plugging is a common feature of airway inflammation and MUC5AC is a central effector of the allergic inflammation required for airway hyperresponsiveness to methacholine.^31^, ^32^ Compared with the controls, asthmatic patients showed increased protein levels of POSTN, SERPINB2, and MUC5AC in their induced sputum supernatant, and PRB1 protein levels in the sputum supernatant were positively correlated with POSTN, SERPINB2, and MUC5AC (*r*_s_ =0.6182, *p* < .0001; *r*_s_ =0.4903, *p* =0.0032, and *r*_s_ = 0.4365, *p* =0.0099, respectively). Therefore, we believe that PRB1 is a potential predictor of type 2‐high asthma.

Our study showed that the expression of PRB1 protein in induced sputum may have reliable diagnostic value for asthma, especially type 2‐high asthma. However, some limitations to our study must be noted. First, in our study, only peripheral blood EOS levels were detected; EOS levels in induced sputum were not detected. Second, we did not conduct research on the underlying mechanism of PRB1 in asthma. Therefore, PRB1 as a potential new therapeutic target requires further study.

## 5 CONCLUSION

The data suggest that PRB1 expression in induced sputum is increased in asthmatic subjects, which demonstrates its association with type 2‐high asthma. Thus, PRB1 may be a biological marker for type 2‐high asthma. The findings of this study could provide new insights into the diagnosis and treatment of asthma.

## CONFLICT OF INTERESTS

The authors declare that there are no conflict of interests.

## ETHICS STATEMENT

The study was approved by the Institutional Research Ethics Committee of the First Affiliated Hospital of Sun Yat‐sen University, and the approval and signed informed consent was obtained from all subjects.

## AUTHOR CONTRIBUTIONS

Feng‐jia Chen, Yu‐xia Liang, and Zhi‐min Zeng conducted the study and collected and analyzed the data. Yu‐biao Guo and Can‐mao Xie designed the study and collected the funds. Li‐juan Du and Chang‐yi Xu collected materials and prepared manuscript. All authors read and approved the final manuscript.

## Supporting information


**Supporting Information**.

## Data Availability

Data are available upon reasonable request.
