# Supplementary figures and images for: Association of increased basic salivary proline‐rich protein 1 levels in induced sputum with type 2‐high asthma
Source: Immun Inflamm Dis. 2022 Mar 10;10(4):e602. doi: 10.1002/iid3.602 (PMC8959441; doi:10.1002/iid3.602)

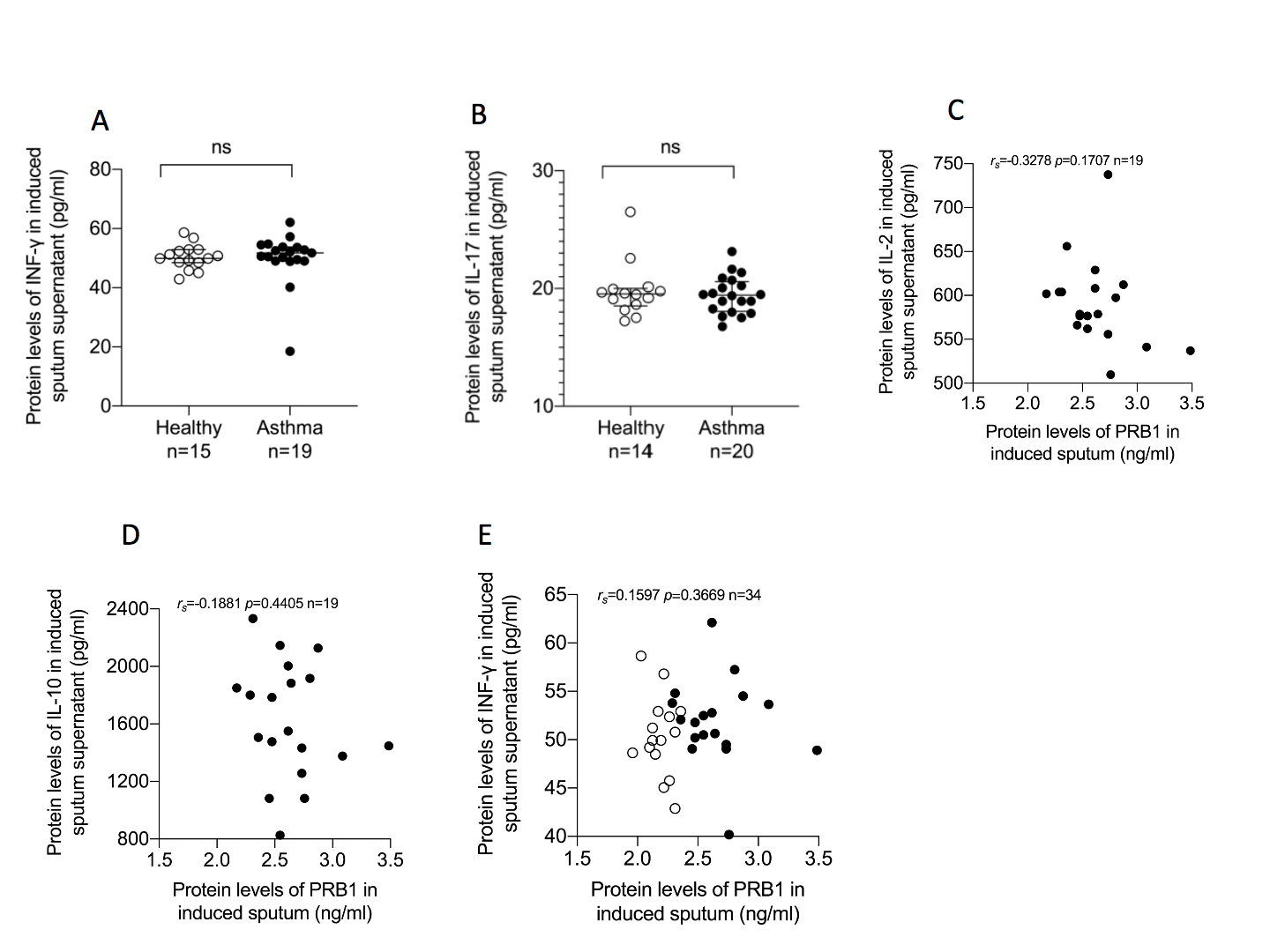

Supplement: Supplementary file 1 — Supporting Information. [file IID3-10-e602-s001.tif]
